# Development and validation of prognostic markers in sarcomas base on a multi-omics analysis

**DOI:** 10.1186/s12920-021-00876-4

**Published:** 2021-01-28

**Authors:** Yongchun Song, Kui Yang, Tuanhe Sun, Ruixiang Tang

**Affiliations:** 1 Department of Oncology Surgery, The First Affiliated Hospital of Xi’an Jiaotong University, 277 West Yanta Road, Xi’an, 710061 Shaanxi China; 2 Department of General Surgery, The First Affiliated Hospital of Xi’an Jiaotong University, Xi’an, 710061 Shaanxi China

**Keywords:** Bioinformatics, Sarcomas, CNV, Methylation, TCGA

## Abstract

**Background:**

In sarcomas, the DNA copy number and DNA methylation exhibit genomic aberrations. Transcriptome imbalances play a driving role in the heterogeneous progression of sarcomas. However, it is still unclear whether abnormalities of DNA copy numbers are systematically related to epigenetic DNA methylation, thus, a comprehensive analysis of sarcoma occurrence and development from the perspective of epigenetic and genomics is required.

**Methods:**

RNASeq, copy number variation (CNV), methylation data, clinical follow-up information were obtained from The Cancer Genome Atlas (TCGA) and GEO database. The association between methylation and CNV was analyzed to further identify methylation-related genes (MET-Gs) and CNV abnormality-related genes (CNV-Gs). Subsequently DNA copy number, methylation, and gene expression data associated with the MET-Gs and CNV-Gs were integrated to determine molecular subtypes and clinical and molecular characteristics of molecular subtypes. Finally, key biomarkers were determined and validated in independent validation sets.

**Results:**

A total of 5354 CNV-Gs and 4042 MET-Gs were screened and showed a high degree of consistency. Four molecular subtypes (iC1, iC2, iC3, and iC4) with different prognostic significances were identified by multiomics cluster analysis, specifically, iC2 had the worst prognosis and iC4 indicated an immune-enhancing state. Three potential prognostic markers (ENO1, ACVRL1 and APBB1IP) were determined after comparing the molecular characteristics of the four molecular subtypes. The expression of ENO1 gene was significantly correlated with CNV, and was noticeably higher in iC2 subtype with the worst prognosis than any other subtypes. The expressions of ACVRL1 and APBB1IP were negatively correlated with methylation, and were high-expressed in the iC4 subtype with the most favorable prognosis. In addition, the number of silent/nonsilent mutations and neoantigens in iC2 subtype were significantly more than those in iC1/iC3/iC4 subtype, and the same trend was also observed in CNV Gain/Loss.

**Conclusion:**

The current comprehensive analysis of genomic and epigenomic regulation provides new insights into multilayered pathobiology of sarcomas. Four molecular subtypes and three prognostic markers developed in this study improve the current understanding of the molecular mechanisms underlying sarcoma.

## Background

Sarcoma is a rare tumor accounting for approximately 1% of all adults but 15% of pediatric malignancies [1, 2]. In 2018, 16,490 cases of soft tissue sarcoma (STS) were diagnosed in the United States, resulting in approximately 6740 deaths [2]. China’s National Central Cancer Registry showed that 28,000 cases of osteosarcoma were newly diagnosed in China in 2015, with an estimated 20,700 deaths [3]. The prognosis of patients with stage IV sarcoma is unfavorable, the median overall survival (mOS) time of STS is about 12 months, and the 5-year survival rate is < 10% [4, 5]. Therefore, it is highly important to identify prognostic biomarkers to improve the accuracy of prognostic prediction and the development of targeted drugs for sarcoma.

The advent of new biochemical techniques, particularly next-generation sequencing, makes it possible to conduct systematic analysis on the genomic characteristics of cancers. Large-scaled, multiomics analyses of various cancers have provided new insights into the dysregulation of cancer genes [6]. Genomic variations caused by the abnormalities of DNA copy numbers (CNVs) and single nucleotide mutations (SNPs) could lead to tumor development [7, 8]. Moreover, epigenetic regulation of DNA methylation on cancer genome also plays a key role in heterogeneous cancer behaviors. Genomic analyses have demonstrated the high heterogeneity of genomic and epigenomic disorders [9–11]. CNV functions critically in sarcoma progression [12, 13]. Transcription disorders resulted from copy number changes are potential driving events in sarcoma progression [14]. In addition, studies of DNA methylation profiles suggested that epigenetic regulation has important biological and clinical significance in sarcoma progression [15–17]. The construction of public, large-scaled and multiomics datasets allow researchers to conduct comprehensive multiomics analysis on the occurrence and development of sarcomas in terms of the effects of genomics, epigenomics, and transcriptomics.

Studies found that the abnormalities of DNA copy number and DNA methylation have important effects on the progression of sarcomas, and the two may also have co-regulatory effects [18, 19]. However, so far, their potential relationship in sarcoma progression has not been studied. In this study, we analyzed DNA copy number, DNA methylation, and related mRNA expressions based on a group of sarcoma patients. Genes with genomic or epigenomic dysregulated expression were identified, and expression correlations between the two types of genes were further analyzed. In addition, molecular subtypes significantly associated with the treatment outcomes of sarcoma were determined by multiomics integration based on genes with genomic and epigenomic dysregulation. Moreover, further systematic analysis was conducted to identify novel mutations with the potential to serve as therapeutic targets or biomarkers for distinguishing sarcoma subtypes. The findings of the present study improve the understanding of the molecular mechanisms of the prognosis of sarcomas.

## Methods

### Data acquisition

The latest clinical follow-up information, CNV, Methylation and RNA-seq data of sarcomas counts were downloaded from TCGA GDC API on 2019.08.14. Meanwhile, the mutect SNV data were obtained from TCGA. A total of 249 samples with the three sets of data were used for subsequent analysis. The GSE21050 and GSE71118 chip data with 286 and 289 samples were downloaded on the Affymetrix Human Genome U133 Plus 2.0 Array from GEO. The details are shown in Table 1.

**Table 1:** Informations of three sets of data set

	TCGA	GSE21050	GSE71118
Metastasis			
No	118	186	186
Yes	119	100	103
Removed	12		
Metastasis (PFS)	*	*	*
No (mean days)	1229.6	1749.51	2500.49
Yes (mean days)	453.6	853.41	793.06
HistologicalType			
DL	57	57	43
UPS	49	128	86
LMS	100	75	78
MFS	23		39
SS	10		
MPNST	8		
DT	2		
Other		26	43
Age			
< 50	51		
>= 50	198		
Gender			
FEMALE	136		
MALE	114		

*DL* dedifferentiated liposarcoma, *UPS* undifferentiated pleomorphic sarcomas, *LMS* leiomyosarcoma, *MFS* myxofibrosarcoma, *SS* synovial sarcoma, *MPNST* malignant peripheral nerve sheath tumors, *DT* desmoid tumors. Other represents an uncertain type other than the 7 types

### Data processing

The expression profile of TCGA-sarcomas data set was preprocessed. Firstly, samples without clinical information or metastatic PFS events < 30 days were removed. Moreover, normal tissue sample data and genes with FPKM equaled to 0 in more than half of the samples were also removed.

Next, the GEO data were preprocessed. Briefly, normal tissue samples were removed only to obtain sarcoma tissues. PFS event data were transformed from year or month to days, followed by mapping microchip probes into human gene SYMBOL using the bioconductor package.

Subsequently CNV intervals were selected and merged according to the following criteria: (1) two intervals with 50% overlapping was considered as a same interval; (2) a CNV with number of covering probes fewer than 5 intervals were removed; (3) the CNV interval was mapped to the corresponding gene using gencode.v22 of GRh38; (4) multiple CNV regions sharing a mutual gene region were merged into one, and the combined CNV values were averaged.

The methylation data were also preprocessed. The missing sites in more than 70% of the samples were removed and replaced by the values calculated by KNN (k-nearest Neighbour) algorithm. Gencode.v22 annotation of GRh38 was used to preserve the probes of the 2 kb upstreams and 200 bp downstreams of the TSS, which were then mapped to the corresponding genes. For SNV data, intron intervals and mutations annotated as silent were removed.

### Identification of CNV-G gene set and MET-G gene set

The Pearson correlation coefficient (r) between each gene with CNV and expression profile (RNA-seq), methylation and expression profile were calculated, respectively, and the correlation coefficient was converted to z-value according to the formula ln ((1 + r)/(1 − r)). Genes with *P* < 1e−5 in correlation coefficient test were taken to construct CNV-significantly correlated gene set (CNV-G) or methylation-significantly correlated gene set (MET-G).

### Identification of molecular subtypes of CNV-G gene set and MET-G gene set

Nonnegative matrix factorization (NMF) is an unsupervised clustering method widely used in analyzing tumor molecular subtypes based on genomics [20, 21]. We applied NMF method was used to cluster the samples based on the expression profiles of the CNV-G and MET-G sets to further examine the relationship between the expression and phenotype of the CNV-G and MET-G sets. Subsequently, the clinical characteristics of the samples and the relationship between their molecular subtypes were analyzed. The standard "brunet" was selected and 50 iterations was performed. The number of clusters k was set between 2 and 10. The average contour width of the common member matrix was calculated by R software package NMF[22], with each subclass consisting a minimum member of 10. The optimal number of clusters was determined according to the indicators such as cophenetic, rss, and silhouette.

### Identification of molecular subtypes

To integrate CNV-G copy number variation (CNV) data, MET-G methylation data (MET) and gene expression profile data (EXP) into CNV-G + MET-G, the "iCluster"[23] method in the R package was used for multi-omics data integration cluster analysis. The optimal data weight values (lambda values) of CNV, MET, and EXP were determined with 20 iterations and 185 lambda of sample points between 0 and 1.

### Relationship between molecular subtypes and tumor microenvironment

TIMER [24] is a network resource for systematic evaluation of the clinical impact of different immune cells on different cancer types. Thus, TIMER was employed to determine the abundance of six immune cell types (B cells, CD4T cells, CD8T cells, neutrophils, macrophages, and dendritic cells) in the microenvironment of sarcomas.

### Genetic analysis of molecular subtypes

The differences of gene expressions in different molecular subtypes were examined. The differentially expressed genes (GEGs) among molecular subtypes were screened by DESeq2 [25] and the qualified DEGs were further identified according to thresholds of the two-fold difference and FDR < 0.05.

### Relationship between molecular subtypes and genomic variation in sarcoma

The differences in genomic variations among molecular subtypes were investigated. Intron and silent mutations were removed from the SNP data downloaded from TCGA. Fisher's precise test was performed to analyze the genes with mutation differences in two-group samples. *P* < 0.05 was the threshold for the detection of genes with mutation differences.

### RT-qPCR

Hs 729 sarcoma cells and 10.014 pRSV-T cells were obtained from ATCC (Manassas, VA, USA). Total RNA from Hs 729 sarcoma cells and 10.014 pRSV-T cells (Control) were extracted by TRIzol (Solarbio, YZ-15596026). According to the instructions of the reverse transcription kit (Thermo, K1622), the RNA was reverse-transcribed into cDNA. Gene expression was determined using SuperReal PreMix (Tiangen, fp204-03) in a real-time PCR system (ABI, 7700). The specific PCR reaction was as follows: pre-denaturated at 95 ℃ for 15 min (min), denaturated at 95 ℃ for 10 s, annealed at 60 ℃ and extended for 32 s. 2^−△△CT^ methods was used to calculate the relative gene expression.

### Comparison with existing sarcomas molecular subtype

According to the literature [26], the molecular subtypes identified in this paper were compared with 7 published molecular subtypes, which were differentiated liposarcoma (DL), leiomyosarcoma (LMS), myxofibrosarcoma (MFS), undifferentiated pleomorphic sarcoma (UPS), synovial sarcoma (SS), malignant peripheral nerve sheath tumor (MPNST), and DT.

### Statistical analysis

The prognostic differences of the subtypes were visualized by Kaplan–Meier (KM). Univariate survival analysis was performed using R package survival, and the prognosis difference test was performed using logrank. Significance was defined when a *P* < 0.05. All of the statistical analyses were performed in R 3.4.3.

## Results

### Comparison of CNVCor and METCor genes

**Fig. 1:**
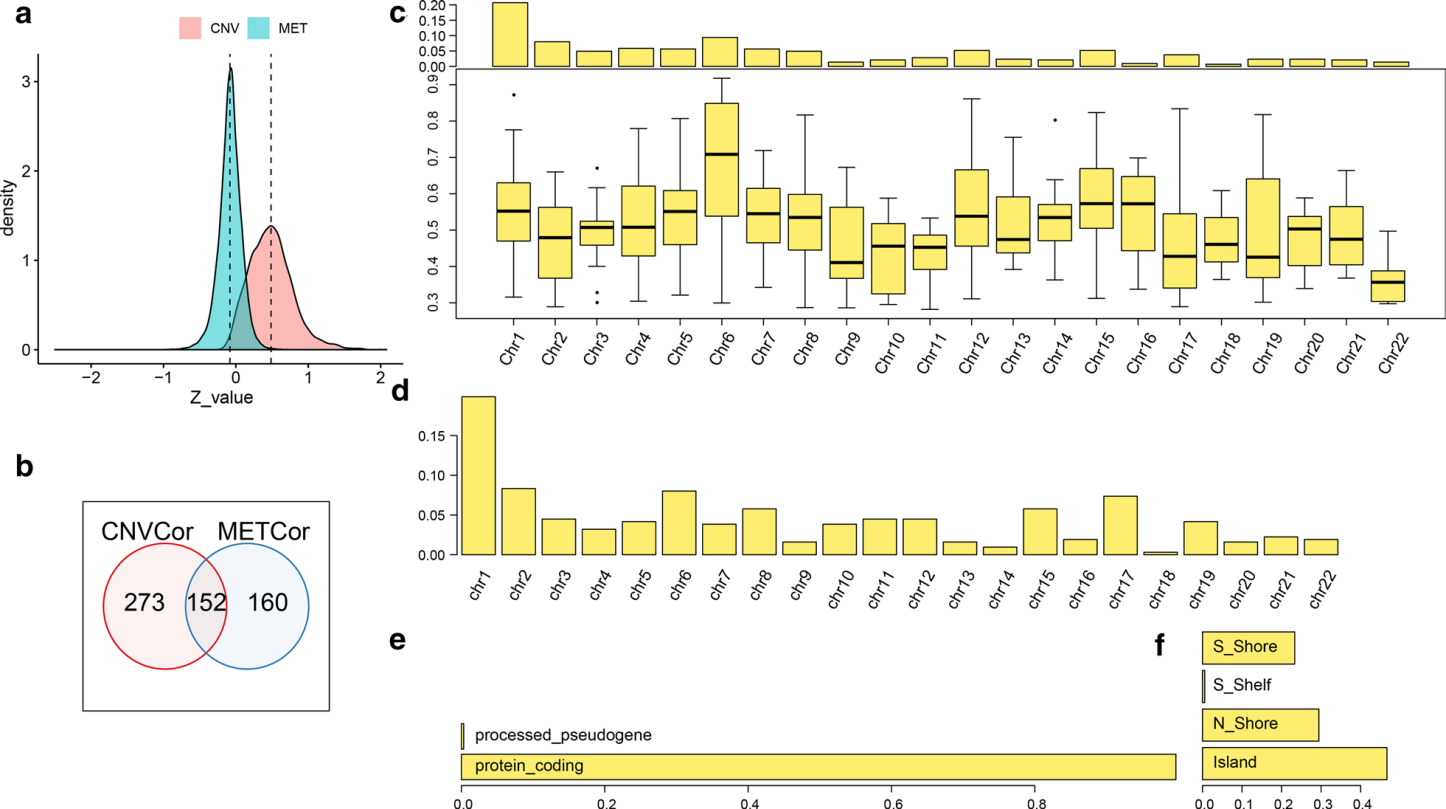
**a** Z-value distribution of correlation between CNVCor and METcor. **b** CNVs and METCor overlap. **c** Distribution of CNVCor on chromosomes (top panel) and correlation (bottom panel). **d** The distribution of METCor on chromosomes. **e** The type of METCor gene. **f** The distribution of MET sites

We obtained a total of 5354 CNVCor genes (correlation between all gene expressions and gene CNV) and 4042 METCor genes (correlation between all gene expressions and gene MET) by conducting correlation analysis with *P* < 1e−5. From the z-value distribution of the two, the correlation of CNVcor significantly shifted to the right, while the correlation of METCor slightly shifted to the left (Fig. 1a). This indicated that CNVcor was mainly positively correlated with gene expressions, while METCor was weakly negatively correlated with genes expressions. Considering the large number of genes in the two gene sets, genes in the CNVCor gene set significantly correlated with new prognosis events (PFS, metastasis/recurrence) and those in the METCor gene set closely correlated with prognosis were selected for subsequent analysis. Here, a resulting 425 CNVCor genes and 312 METCor genes were obtained. A comparison of CNVCor and METCor gene sets further identified 152 overlapping genes (Fig. 1b), suggesting that CNVCor and METCor may be mutually exclusive. According to the distribution of CNVCor and METCor on chromosomes, there was a significant bias in the distribution of CNVCor gene on chromosomes 1, 2, 5 and 6 (Fig. 1c), and METCor exhibited an obvious bias on chromosome 1 (Fig. 1d). METCor mainly contained protein-coding genes (Fig. 1e), and MET loci was largely distributed at the CpG island interval (Fig. 1f).

### Molecular subtypes were identified based on CNVcor and METcor genes

**Fig. 2:**
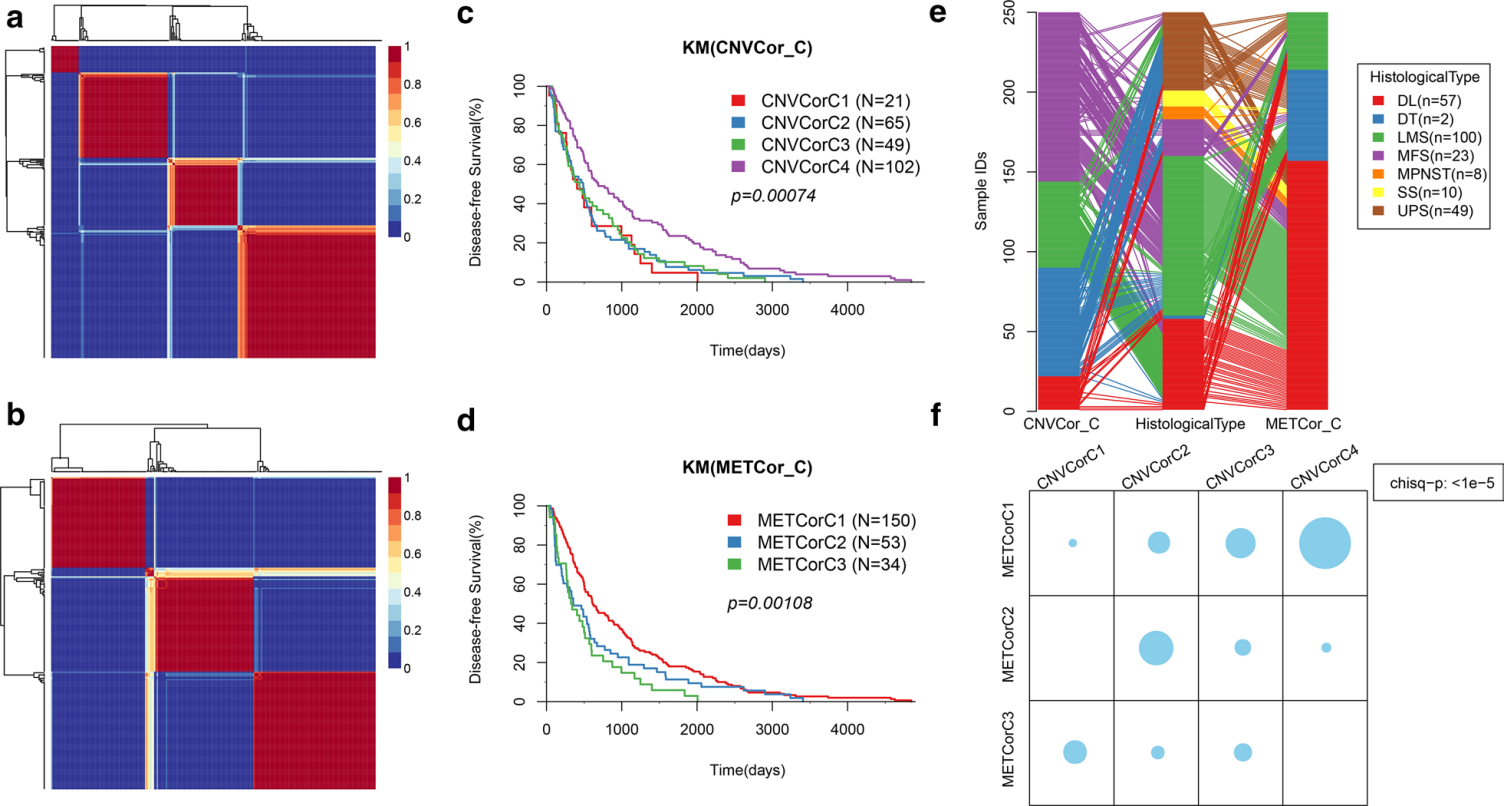
**a** NMF clustering results of CNVCor. **b** NMF clustering results of METCor. **c** KM survival curve of CNVCor subtype. **d** KM survival curve of METCor. **e** The correlation between subtypes of CNVCor cluster, subtypes of METCor cluster and pathological subtypes. **f** The overlap between subtypes of CNVCor cluster and subtypes of METCor cluster

According to the results of NMF, the optimal cluster number of CNVCor was 4 (Fig. 2a), while the optimal cluster number of METCor was 3 (Fig. 2b). There were significant differences in the sample PFS survival curves between the four subclasses of CNVCor (Fig. 2c), moreover, similar results were also detected from the survival curves of METCor subclasses (Fig. 2d). Analysis of the relationship among CNVCor, METCor subtypes, and pathological subtypes showed that CNVCor_C1 mainly corresponded to METCor_C3 and included multiple pathological subtype samples; CNVCor_C2 mainly corresponded to METCor_C3 and METCor_C1 and included DL, LMS, and MFS subtypes; CNVCor_C3 mainly corresponded to METCor_C2, included UPS subtypes and some LMS subtypes; CNVCor_C4 was composed of multiple pathological subtypes (Fig. 2e). Noticeably, there was a significant overlapping of the four subclasses of CNVCor with the three subclasses of METCor (Fig. 2f).

### CNV, MET, and EXP data were integrated to cluster samples to determine molecular subtypes

**Fig. 3:**
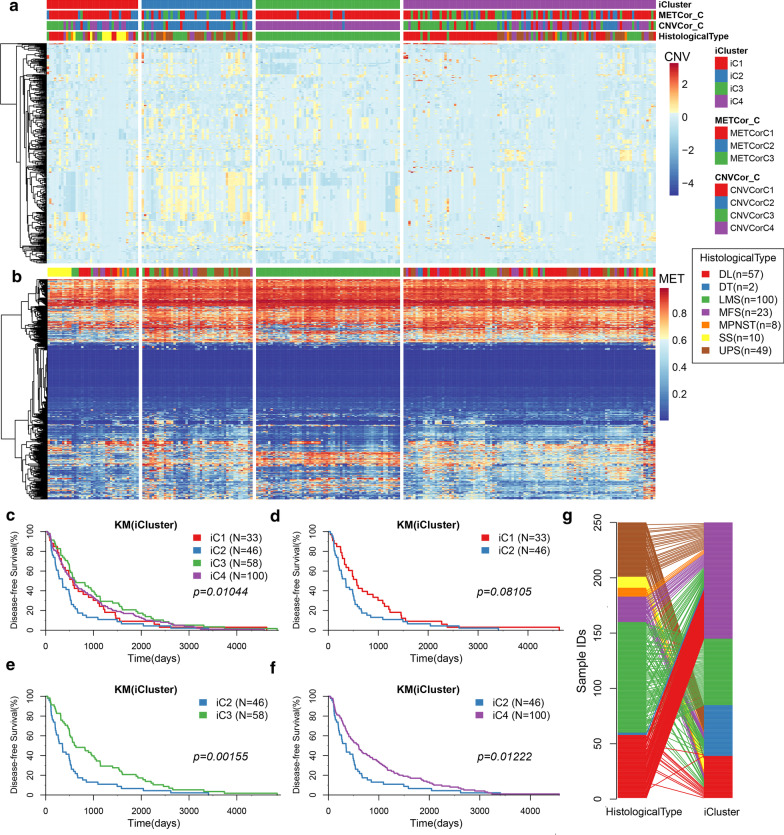
**a** Heatmap of the expression of subtype CNVCor identified by iCluste. **b** Heatmap of the expression of subtype METCor identified by iCluste. **c** The PFS KM curve between the subtypes identified by iCluster. **d** PFS survival curve between iC1 and iC2 subtypes. **e** PFS survival curve between iC2 and iC3 subtypes. **f** PFS survival curve between iC2 and iC4 subtypes. **g** The corresponding relationship between iC subtype and pathological subtype

By clustering multiple omics data, four subclasses (iC1, iC2, iC3, and iC4) (Fig. 3a, b) with significantly different PFSs were determined (Fig. 3c). The prognosis of the iC2 subtype with the worst prognosis was compared with the other three subtypes, and we found that the prognosis of iC2 showed the greatest differences with iC3 and iC4 subtypes, and there was no significant difference between iC2 and iC1 (Fig. 3d–f). Prognostic analysis of OS of the four subtypes also showed significant differences (Additional file 1: Figure S1). Comparison between iC subtypes and pathological subtypes revealed that the iC2 with the worst prognosis was related to most UPS and some LMS subtypes, while iC3 was almost entirely composed of LMS subtypes, and iC4 was composed of most DL, MFS and a few UPS and LMS subtypes (Fig. 3g).

### Abnormalities of DNA copy number were consistent with methylation abnormalities

**Fig. 4:**
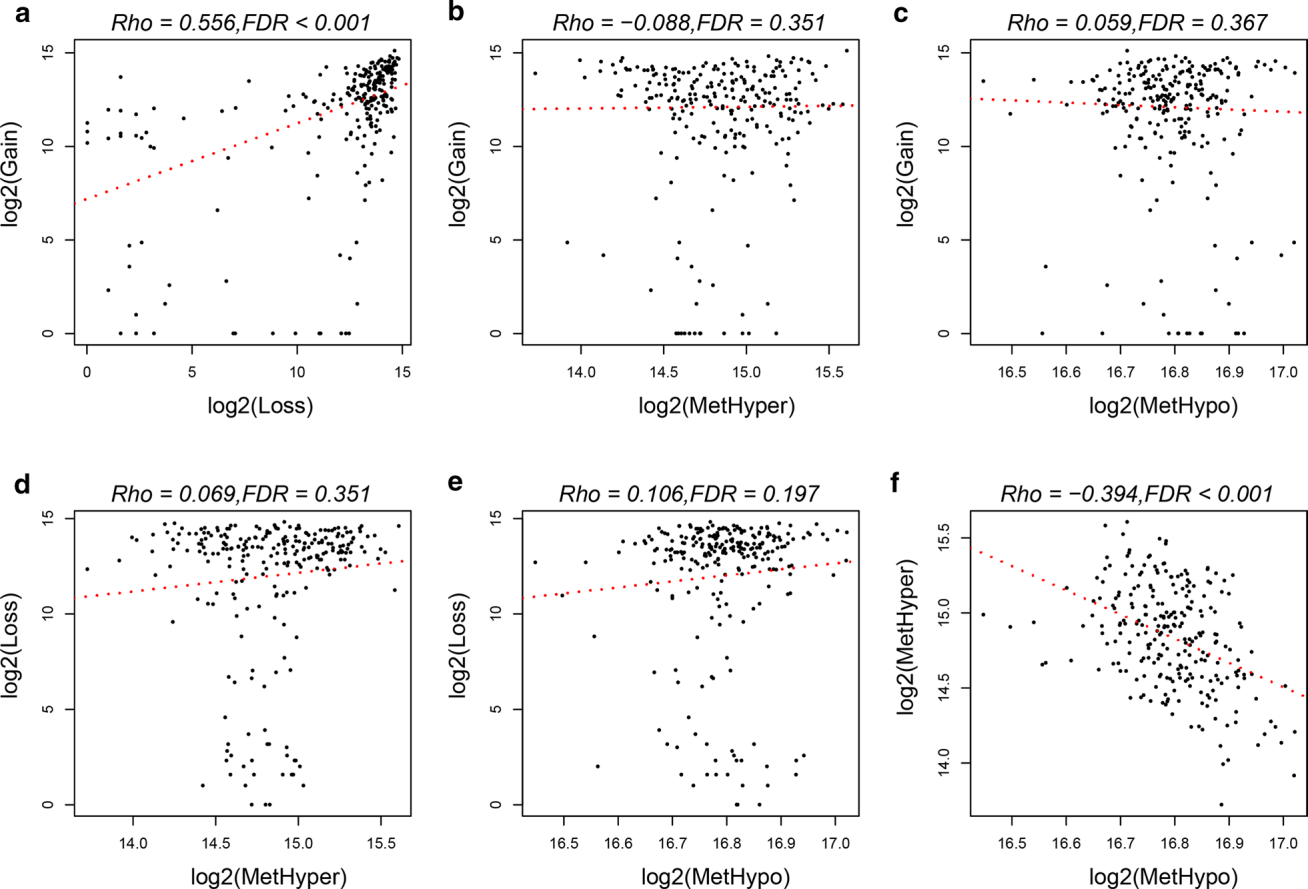
**a** Frequency distributions of Gain and Loss in CNV. **b** Frequency distributions of Gain and MetHyper. **c** Frequency distributions of Gain and MetHypo. **d** Frequency distributions of CNV Loss and MetHyper. **e** Frequency distributions of CNV Loss and MetHypo. **f** Frequency distributions of MetHyper and MetHypo

To study the relationship between CNV and MET anomalies, β-value of CNV > 0.3 was defined as CNV Gain, while β-value < 0.3 was defined as Loss, β value of MET > 0.8 was defined as MetHyper, while the β value of MET < 0.2 was defined as MetHypo. Our data revealed a significant correlation between CNV Gain and Loss (Fig. 4a). However, no significant correlation was observed between Gain and MetHype/MetHypo (Fig. 4b, c) or between Loss and MetHyper/MetHypo (Fig. 4d, e). MetHyper was found to be significantly negatively correlated with MetHypo (Fig. 4f).

### Immune score of sarcoma subtypes

**Fig. 5:**
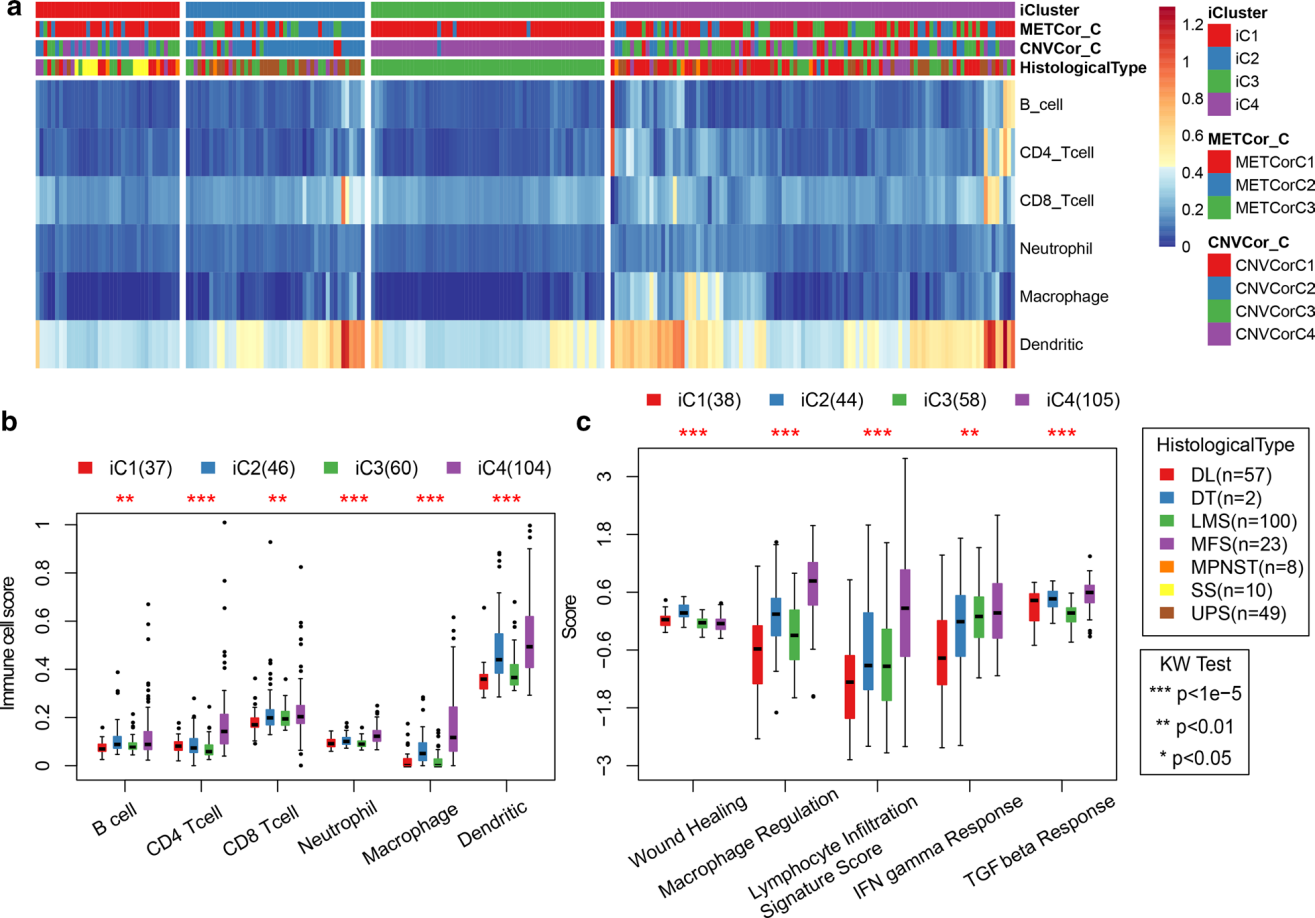
**a** The scores of six immune cells in all the samples. **b** The scores of the six immune cells were in the three subtypes of iCluster. **c** Immunosignature scores of 5 types

Clinical differences such as gender and age of iC2 and iC1/iC3/iC4 subtypes exhibited great differences, especially the Age samples, which were noticeably more than 60 in the iC2 subtype (Table 2). Furthermore, Tumor Immune Estimation Resource (TIMER) tool was employed to compare the immune scores of four subtypes, and the six types of immune cells in the iC4 subtype with the most favorable prognosis scored higher than the other subtypes (Fig. 5a). Moreover, the proportion of the six immune cells in the iC4 subtype was significantly higher than that of other subtypes (Fig. 5b). These data suggested that the iC4 subtype may be in an immune-enhanced state. Moreover, comparisons of lymphocyte infiltration score, IFN gamma response score and TGF beta response score of iC4 subtype were significantly higher than other subtypes (Fig. 5c).

**Table 2:** Clinical features between SARC subtypes

Clinical features	Total	iC2	iC1/iC3/iC4	*P* value
Event				0.27
Alive	155	23	132	
Dead	94	23	71	
Age				0.0134
0–50	51	8	43	
50–60	65	9	56	
60–70	67	10	57	
70–80	44	11	33	
80–100	22	8	14	
Gender				0.33
Female	135	26	109	
Male	114	20	94	
HistologicalType				0.203
DL	57	2	55	
DT	2		2	
LMS	100	14	86	
MFS	23	6	17	
MPNST	8	1	7	
SS	10		10	
UPS	49	23	26	
NewEventType				0.357
DistantMetastasis	67	19	48	
LocoregionalDisease	1		36	
LocoregionalRecurrence	48	4	14	
NewPrimaryTumor	8	2	1	
Primary	125	21	104

### Molecular characteristics of the four sarcoma subtypes

**Fig. 6:**
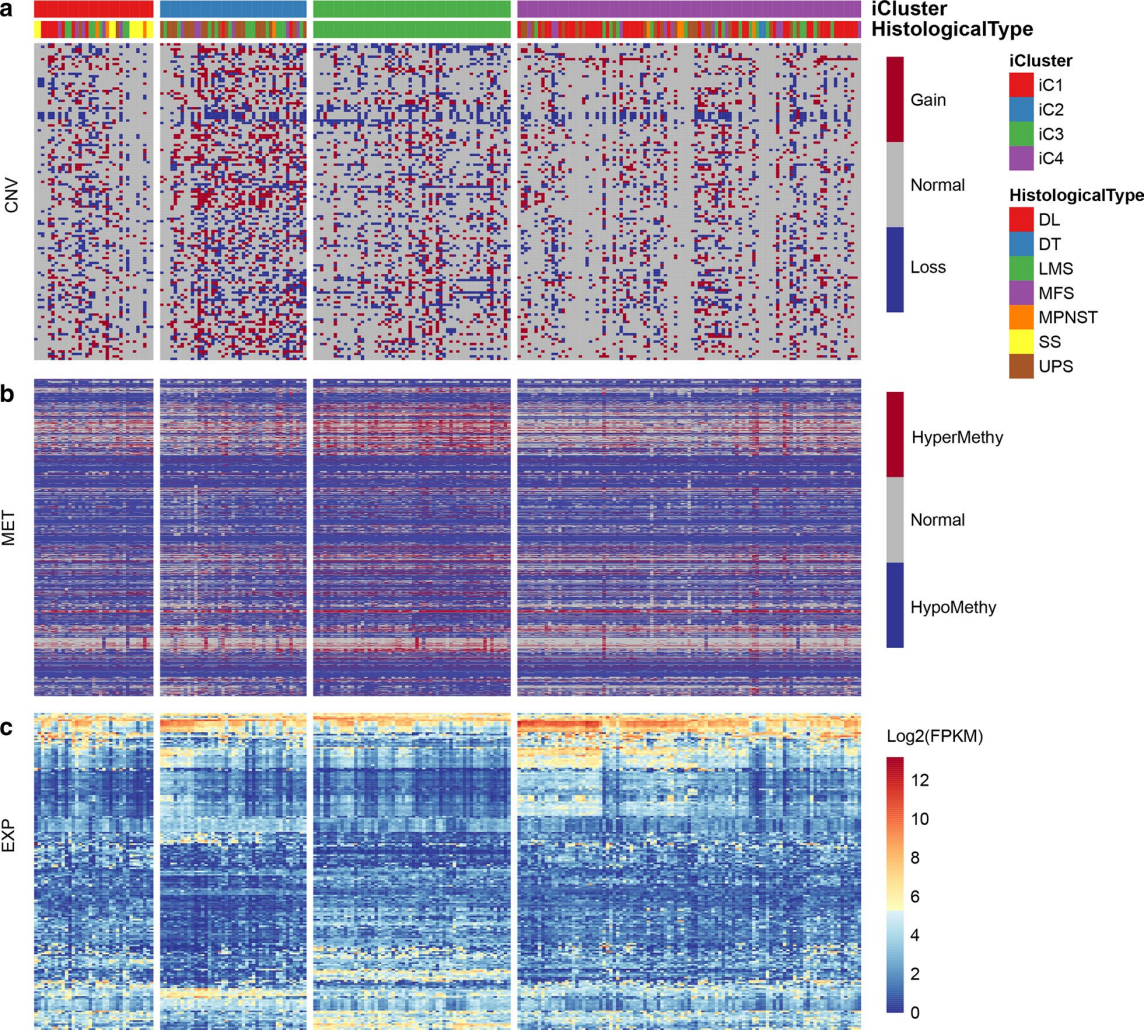
**a** Distribution of CNV in the iCluster subtype. **b** Distribution of methylation in the iCluster subtype. **c** Heatmap of differentially expressed genes in iCluster subtypes

Based on the results of iCluster, gene expression differences between iC1 and iC2/iC3/iC4 subtypes with significant prognostic differences were compared, and a total of 604 DEGs were obtained after removing low-expressed genes. The GO enrichment analysis indicated that those genes were noticeably enriched to cell adhesion, inflammation response, mesenchyme development and metastasis and immune response (Additional file 2: S2, Additional file 3: Figure S3A). The CNV frequency of 604 DEGs in iC2 was greatly higher than that of iC1, iC3 and iC4, suggesting that CNV had certain effects on the prognosis of SARC (Fig. 6a). However, no significant difference in methylation of the four subtypes was detected (Fig. 6b). After analyzing the correlation between gene expression, methylation, and CNV, we found that DEGs were high-expressed in demethylated samples (Fig. 6c), but this was not observed in CNV, suggesting that the effect of methylation on the expressions of DEGs was stronger than CNV.

**Fig. 7:**
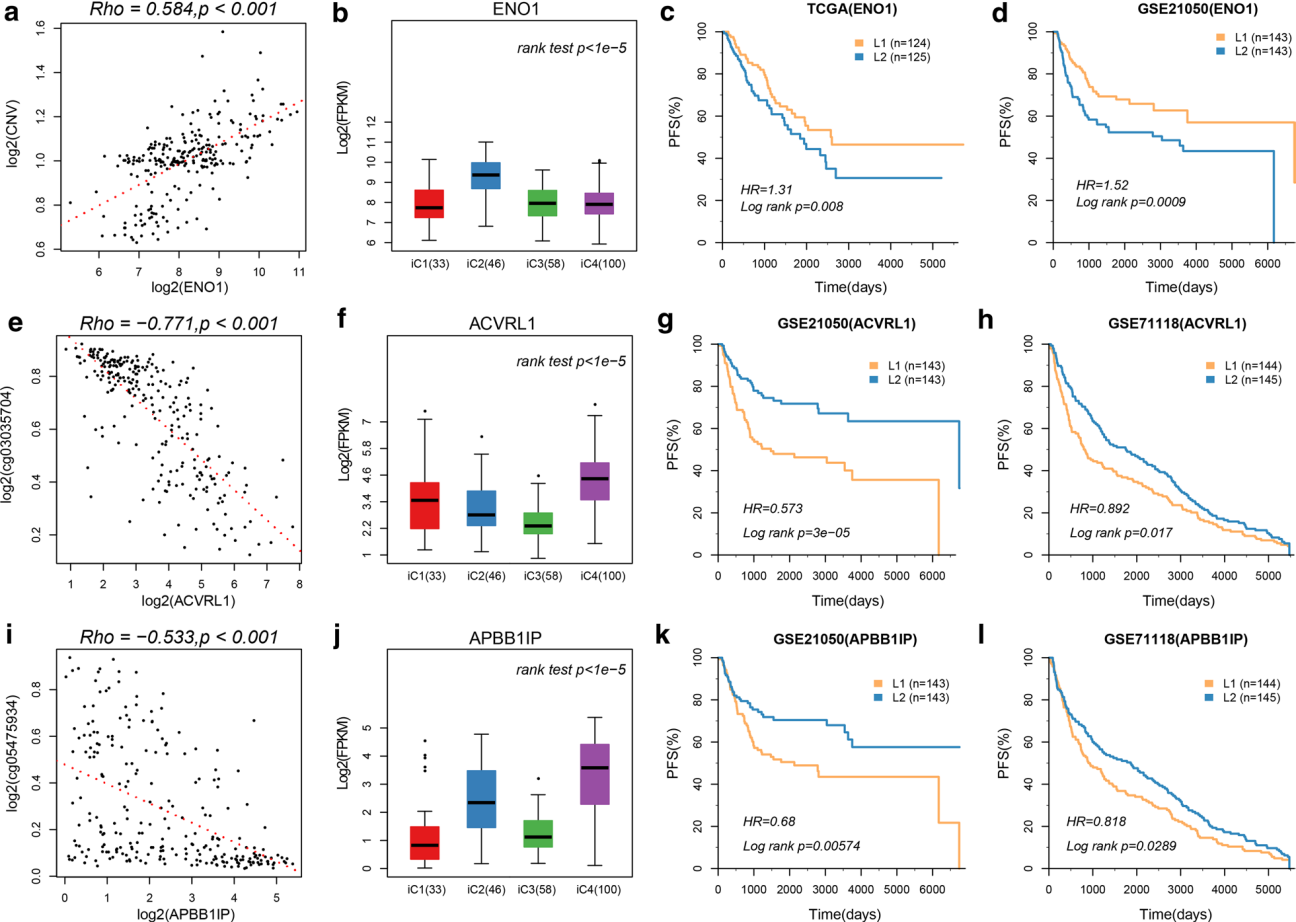
**a**–**d** Correlation between NO1 gene methylation and expression, expression of NO1 in iC subtype, OS KM curve of samples from high-expression group and low-expression group in TCGA data, and OS KM curve of samples from high-expression group and low-expression group of GSE21050 verification set. **e**–**h** Correlation between ACVRL1 gene methylation and expression, expression of ACVRL1 in iC subtype, OS KM curve of samples from high-expression group and low-expression group of GSE21050 data and GSE7118 data. **i**–**l** Correlation between APBB1IP gene methylation and expression, expression of APBB1IP in iC subtype, OS KM curve of samples from high-expression group and low-expression group of GSE21050 data and GSE7118 data

To investigate the relationship between gene expressions and CNV/MET, 604 DEGs were used for prognostic survival analysis. Univariate survival analysis determined 127 genes significantly associated with sarcoma prognosis, among them 41 gene were closely associated with PFS in at least two data sets (Additional file 3: Figure S3B). Three of the most significant genes, namely, ENO1, ACVRL1 and APBB1IP, were further used to analyze the relationship of their expressions and CNV and methylation. It has been found that the expression of ENO1 gene was positively correlated with CNV (Fig. 7a), noticeably, ENO1 expressed significantly higher in the iC2 subtype with the worst prognosis than in other subtypes (Fig. 7b). Correspondingly, high-expressed ENO1 was associated with worse prognosis recorded in TCGA and GSE21050 (Fig. 7c, d). The expressions of ACVRL1 and APBB1IP were negatively correlated with methylation (Fig. 7e–i), moreover, the two genes exhibited high expressions in the iC4 subtype with the most optimal prognosis (Fig. 7f–j), and their high expressions were related to a better prognosis (Fig. 7g, h, k, l). RT-qPCR assay results indicated that ENO1 was upregulated, whereas ACVRL1 and APBB1IP were downregulated in Hs 729 sarcoma cells that in 10.014 pRSV-T cells (Additional file 4: Figure S4).

### Mutation spectrum of the four sarcoma subtypes

**Fig. 8:**
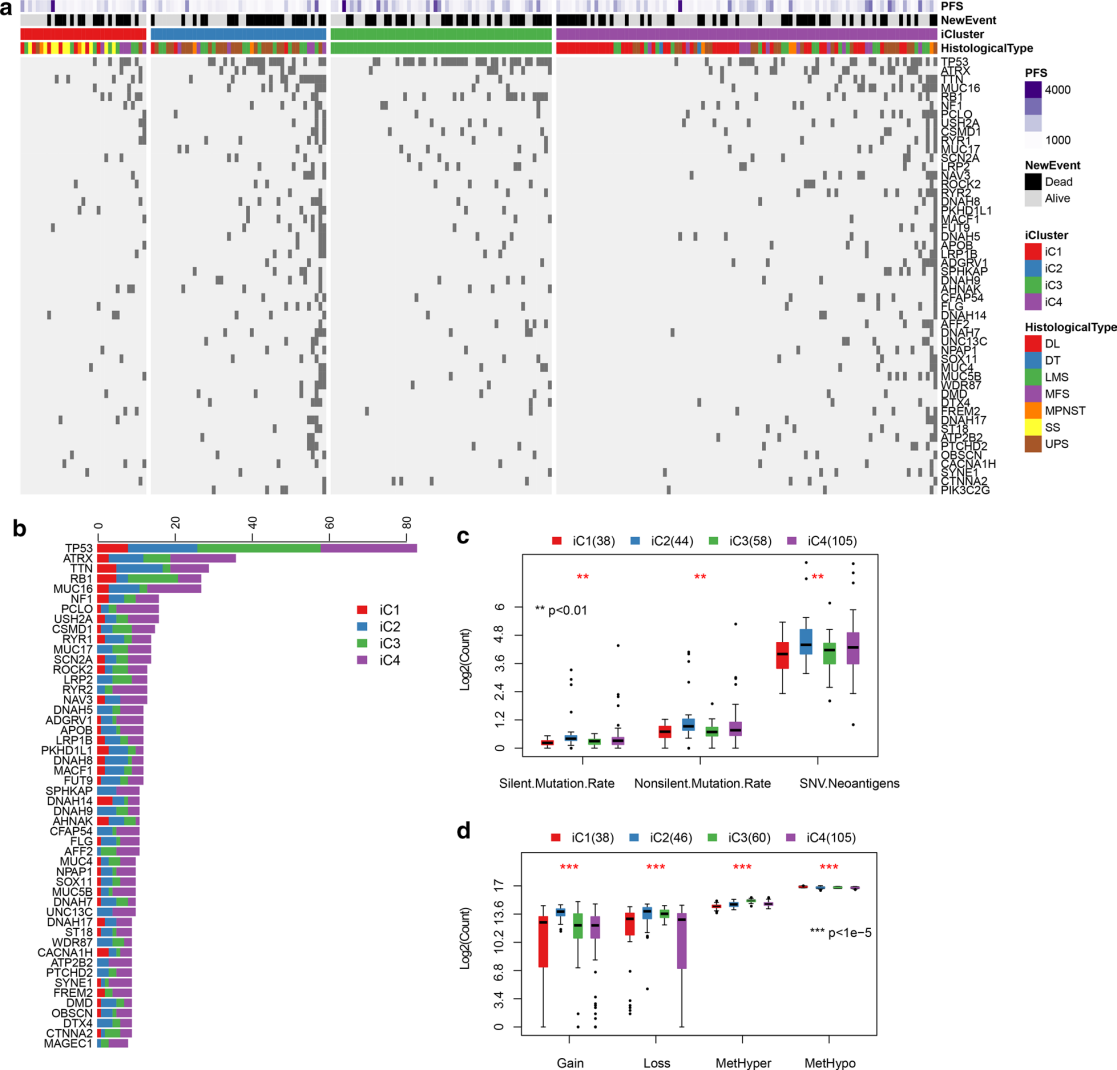
**a** The top 50 mutated genes between iC subtypes. **b** The number of mutations of the top 50 genes in the iC subtype. **c** Distribution of silent, nonsilent and neoantigens on iC subtypes. **d** Distribution of CNV Gain/Loss and methylated MetHyper/MetHypo on iC subtypes

The mutation spectra of different subtypes were analyzed to select the top 50 genes (Fig. 8a, b). By analyzing the mutant spectrum differences, we found that the number of silent/nonsilent mutations and neoantigens in the iC2 subtype was significantly higher than that in iC1/iC3/iC4 subtype (Fig. 8c). The same trend was also observed in the number of Gain/Loss of CNV (Fig. 8d). These results indicated that genomic instability had an important impact on the prognosis of sarcoma because subtypes with high mutation rate and high CNV were related to worse prognosis. Although there were significant differences of MetHyper/MetHypo in the four subtypes, iC2 did not show a significant difference when compared with other three subtypes (Fig. 8d).

### The relationship of molecular subtypes and markers with previously developed subtypes and markers

**Fig. 9:**
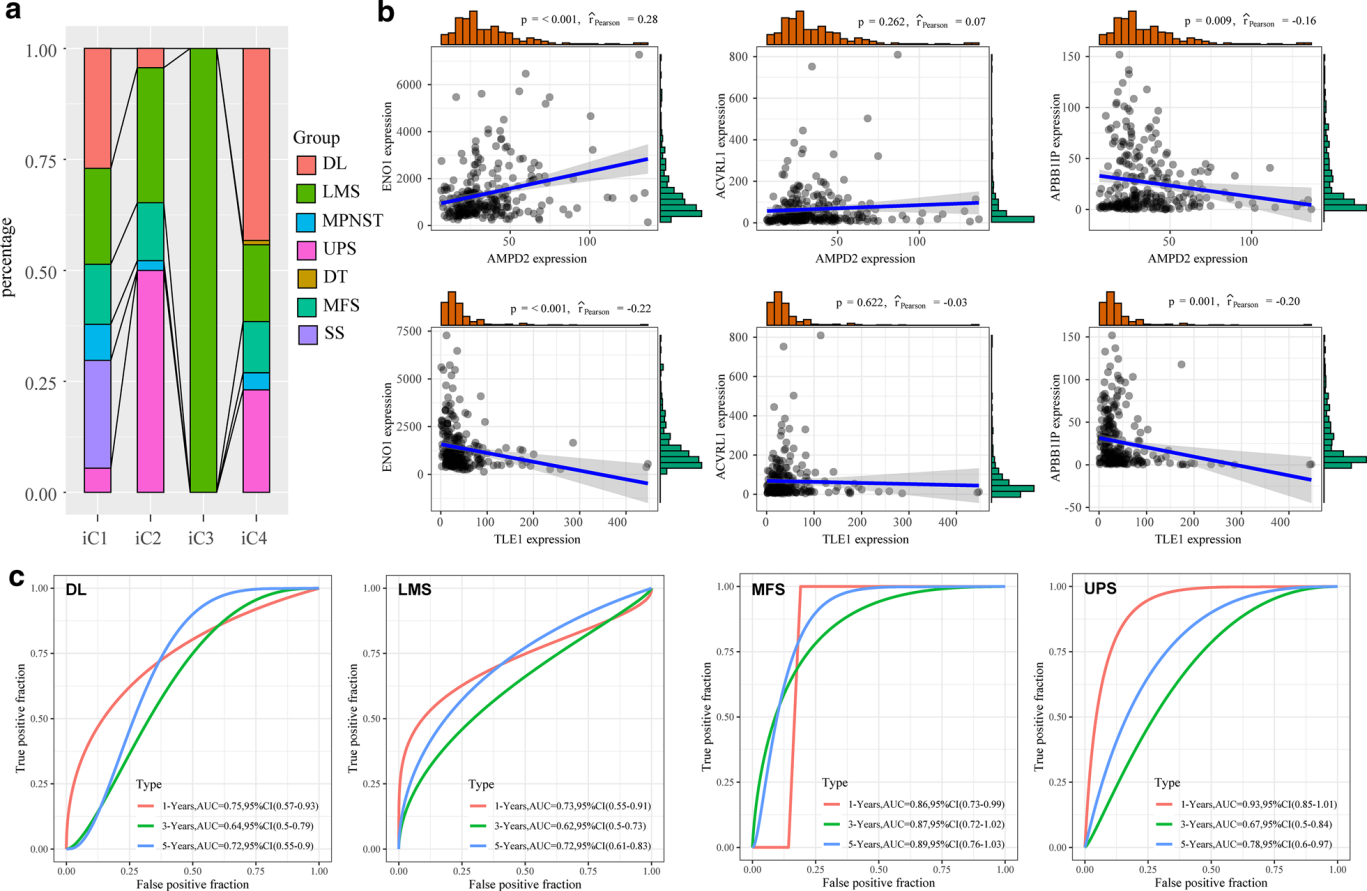
**a** Distribution of 7 histological types in four molecular subtypes. **b** The expression relationship between AMPD2/TLE2 genes and three potential prognostic markers (ENO1, ACVRL1 and APBB1IP). **c** Prognostic ROC curves of three potential Prognostic Models (ENO1, ACVRL1 and APBB1IP) in four categories of samples (DL, LMS, MFS and UPS)

We analyzed the relationship among the four molecular subtypes and the seven different histological types according to the WHO classification (Fig. 9a), and significant differences among the seven different histological types were detected from the four molecular subtypes. Specifically, IC3 subtypes were all LMS, while DL and UPS were significantly enriched in iC4 subtype and iC2 subtypes, respectively. These results indicated that the three subtypes were closely correlated with histological types, moreover, the absence of typical histological type enrichment to iC1 subtype suggested the possible existence of a new molecular type.

The expression of the transducin-like enhancer of split-1 (TLE1) serves as a biomarker for the diagnosis of synovial sarcoma. Herein, we analyzed the relationship between TLE1 gene and three potential prognostic markers (ENO1, ACVRL1 and APBB1IP) by determine their expressions. The results showed that TLE1 was significantly negatively correlated with ENO1, and positively correlated with APBB1IP (Fig. 9b). Study indicated that high-expressed AMPD2 is correlated with poor prognosis of UPS [27]. The correlation between AMPD2 expression and three potential prognostic markers (ENO1, ACVRL1 and APBB1IP) was also investigated, and our results showed that TLE1 was significantly positively correlated with ENO1, and was negatively correlated with APBB1IP. In our study, both high-expressed ENO1 and low-expressed APBB1IP were predictive of a poor sarcoma prognosis, suggesting that the three potential prognostic markers were closely associated with known genetic markers.

Furthermore, the three potential prognostic markers (ENO1, ACVRL1 and APBB1IP) in predicting the sarcoma prognosis of different histological types were examined, due to the sample sizes of DT, MPNST, and SS, the prognostic role of the three genes were analyzed in DL, LMS, MFS and UPS. A 3-gene signature was established by multifactorial survival analysis to calculate the risk score of the samples, and the performance of the signature in predicting the prognosis of DL, LMS, MFS, and UPS was respectively examined. We observed that the 3-gene signature was significantly more accurate in predicting short-term survival rather than long-term survival. Moreover, it showed a better predictive performance in MFS and UPS patients, especially in MFS, with AUCs of more than 0.86 of 1-, 3-, and 5-year survival (Fig. 9C).

## Discussion

Genomic instability, which is a hallmark of malignancy, could lead to DNA copy number changes in most cancer types [28, 29]. In viral infection and alcohol-related liver cancer, amplification of the common chromosome 8q24 copy number could cause high expression of the oncogene c-myc, indicating that the development of some liver cancers may be c-myc-dependent [30]. In early HCC, common 1q21 amplification and heterozygosity loss (LOH) of 1p36 and 17p13 suggest that these chromosomal aberrations may induce HCC [31]. In addition to copy number abnormalities, DNA methylation is an important regulator of gene transcription and one of the most widely studied epigenetic modifications. Hypermethylation of tumor suppressor gene CpG island is commonly detected in tumors and is largely tumor-specific [32, 33]. For example, hypermethylation of the BRCA1 gene CpG island mainly occurs in breast and ovarian cancer, while hypermethylation of the hMLH1 gene is commonly found in colon, stomach and endometrial cancers. A comprehensive analysis of the multilayered genomic characteristics of cancer could identify molecular subtypes and help candidate therapeutic targets and biomarkers. This study analyzed the relationship between epigenetics and CNV, and observed that the abnormalities of DNA copy number and methylation shared a mutual consistency. Based on multi-omics association analysis, CNV-G and MET-G gene sets were identified, and the relationship between CNV and methylation was established with the gene expressions. Finally, by performing multi-omics clustering based on gene expression, DNA methylation, and CNV, four molecular subtypes (iC1, iC2, iC3, iC4) with difference in age (Additional file 5: Figure S5) were determined, noticeably, iC2 was associated with poor clinical results. In addition, we also identified 3 gene prognostic markers and verified them in external data sets.

A growing number of studies have shown that tumor-infiltrating lymphocytes (TILs) are involved in tumor progression and invasion. TIL consists of a variety of lymphocytes with different activities, the most common lymphocytes are CD8+ and CD4+ T cells [34]. T-lymphocyte infiltration of primary tumors is used to predict clinical outcomes of cancers such as breast cancer [35], head and neck cancer [36], non-small-cell lung cancer [37], colorectal cancer [38] and gastric cancer [39]. In our study, there were also significant differences in the immune microenvironment of the four molecular subtypes, but the iC4 subtype with the optimal prognosis showed higher scores of the six immune cells than the other subtypes, especially, its scores of lymphocyte infiltration, IFN gamma response and TGF beta response were significantly higher than the other subtypes. These results indicated that the iC4 subtype may be in an immune enhanced state.

The molecular characteristics of the four molecular subtypes were compared, and we determined three genes, namely, ENO1, ACVRL1 and APBB1IP, which were closely associated with the prognosis of sarcoma. ENO1 gene, which had a significant positive correlation with CNV, was high-expressed in iC2 subtype and served as a risk factor. The expression of ACVRL1 and APBB1IP was negatively correlated with the degree of methylation, and the two genes with high expressions in iC4 subtype was two protective factors. The role of eno1enolase (enolase-1) in non-small cell carcinoma has been widely studied by previous researches. Chang et al. found significantly downregulated expression of ENO1 in NSCLC compared with normal tissues, and indicated that low-expressed ENO1 may suggest a favorable prognosis [40]. Zhang et al. [41] confirmed that the expression of ENO1 protein in NSCLC tissues and plasma was greatly higher than that in patients with benign tumors. ENO1 was less studied in sarcomas. Takahashi A et al. found that ENO1 showed obvious differential expression in histological subtypes of sarcoma [42]. ACVRL1, also known as activin receptor-like kinase 1(ALK1), is a direct receptor for TGF. ALK1 determines the characteristics of vascular endothelial cells and plays an important role in angiogenesis [43]. Study demonstrated that ALK1 could promote endothelial cell proliferation after activation and then stimulate tumor blood vessel formation, which is essential to tumor development and metastasis [44]. Cunha et al. investigated ALK1 receptor on the surface of vascular cells, and observed that tumor angiogenesis in mice is inhibited and the tumor grows slowly when the expression of ALK1 receptor is inhibited [45, 46]. APBB1IP is alternatively known as RIAM, silencing RIAM in melanoma cells leads to the inhibition of tumor growth and hinders metastasis in a mouse xenograft model combined with immunodeficiency [47]. These results indicated that the three genes could be clinically applied to treat sarcoma, and may provide a potential target for predicting the prognosis of clinical patients with sarcoma.

Although the relationship between epigenetics and genomic variations has been systematically analyzed by bioinformatics techniques, some limitations of this study should still be noted. Firstly, our samples lacked some clinical follow-up information, thus, factors such as the presence of other health conditions of the research subjects were not considered. Also, the results obtained only by bioinformatics analysis were not convincing enough, which therefore requires experimental verifications to confirm the present results. Moreover, further genetic and experimental studies involving larger sample sizes and experimental validation are needed.

## Conclusion

In summary, in this study, we analyzed the possible pathogenesis of sarcomas through multi-omics analysis of genomics, epigenomics, and transcriptomics, and discovered that DNA CNV and methylation play important roles in sarcomas. In addition, we identified 4 potential molecular subtypes of sarcoma and 3 key biomarkers. These novel mechanisms and clinical classifications may facilitate a more accurate diagnosis and targeted therapy for sarcoma patients.

## Supplementary Information

**Additional file 1: Supplementary Figure 1**. Prognostic analysis of OS of the four subtypes. A: KM survival curve of four subtypes. B: KM survival curve between iC1 and iC2. C: KM survival curve between iC2 and iC3. D: KM survival curve between iC2 and iC4.

**Additional file 2: Supplementary Figure 2**. Functional enrichment analysis. A: The GO enrichment analysis. B: The KEGG enrichment analysis.

**Additional file 3: Supplementary Figure 3**. Functional enrichment analysis and identification of differentially expressed genes. A: Heatmap of The GO enrichment analysis. B: Differentially expressed genes in three datasets.

**Additional file 4: Supplementary Figure 4**. Differential expression of three genes (ENO1, ACVRL1 and APBB1IP) were determined by RT-qPCR.

**Additional file 5: Supplementary Figure 5**. Age in iC1 subgroup is significantly lower than iC2 and iC4, and age in iC3 is significantly lower than iC4.

## Data Availability

The data that support the findings of this study are openly available in TCGA-SARC (https://portal.gdc.cancer.gov/projects/), and GEO, reference number GSE21050 (https://www.ncbi.nlm.nih.gov/geo/query/acc.cgi?acc=GSE21050) and GSE71118 (https://www.ncbi.nlm.nih.gov/geo/query/acc.cgi?acc=GSE71118).

## Abbreviations

TCGA: The Cancer Genome Atlas
MET-G: Methylation-related genes
CNV-G: CNV abnormality-related genes
SNPs: Single nucleotide mutations
mOS: Median overall survival
STS: Soft tissue sarcoma
NMF: Nonnegative matrix factorization
ALK1: Activin receptor-like kinase
EXP: Gene expression profile data
KNN: K-nearest neighbour
TIMER: Tumor immune estimation resource
