# Supplementary figures and images for: Development and validation of prognostic markers in sarcomas base on a multi-omics analysis
Source: BMC Med Genomics. 2021 Jan 28;14:31. doi: 10.1186/s12920-021-00876-4 (PMC7841904; doi:10.1186/s12920-021-00876-4)

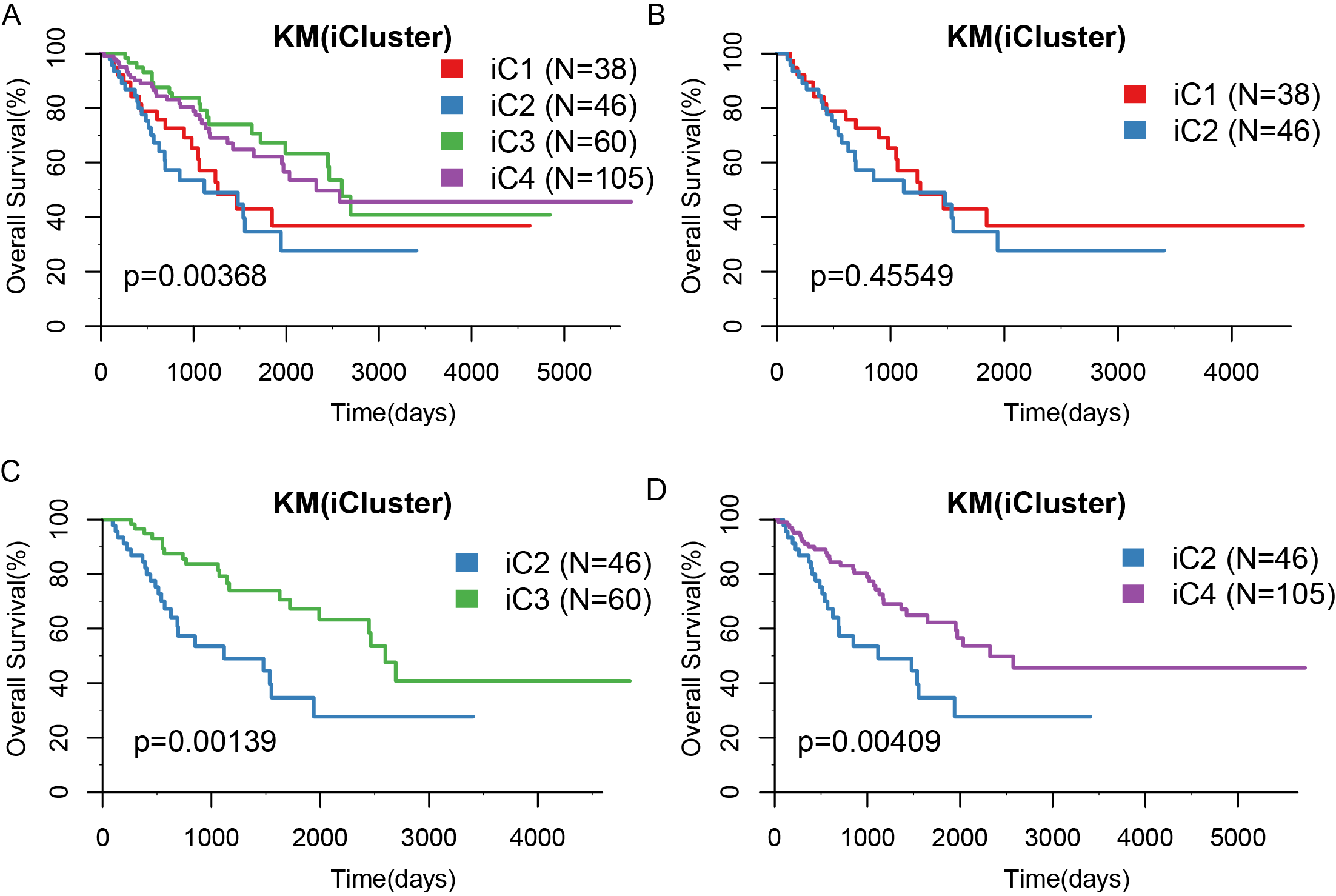

Supplement: Supplementary file 1 — Additional file 1: Supplementary Figure 1. Prognostic analysis of OS of the four subtypes. A: KM survival curve of four subtypes. B: KM survival curve between iC1 and iC2. C: KM survival curve between iC2 and iC3. D: KM survival curve between iC2 and iC4. [file 12920_2021_876_MOESM1_ESM.tif]

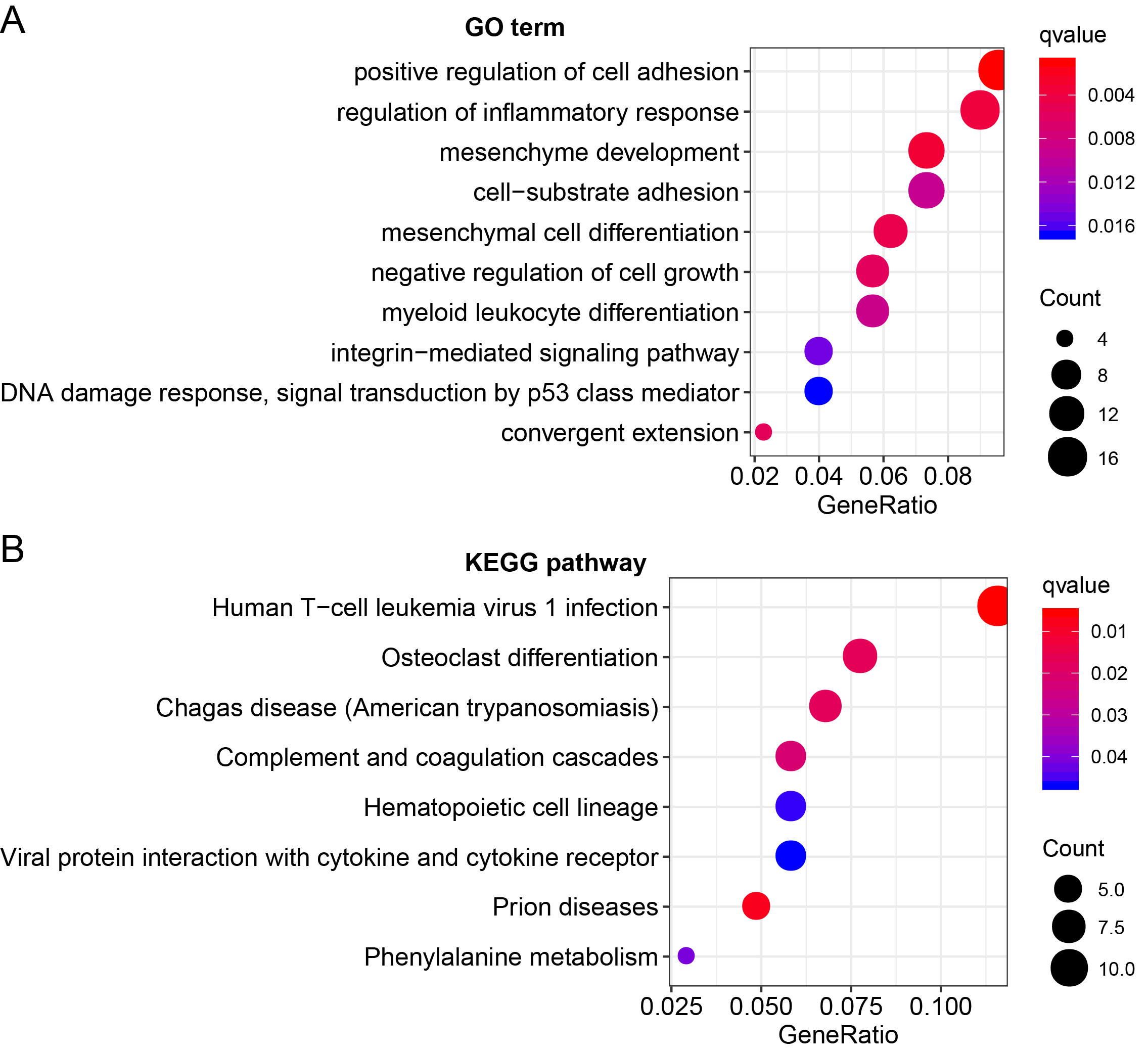

Supplement: Supplementary file 2 — Additional file 2: Supplementary Figure 2. Functional enrichment analysis. A: The GO enrichment analysis. B: The KEGG enrichment analysis. [file 12920_2021_876_MOESM2_ESM.tif]

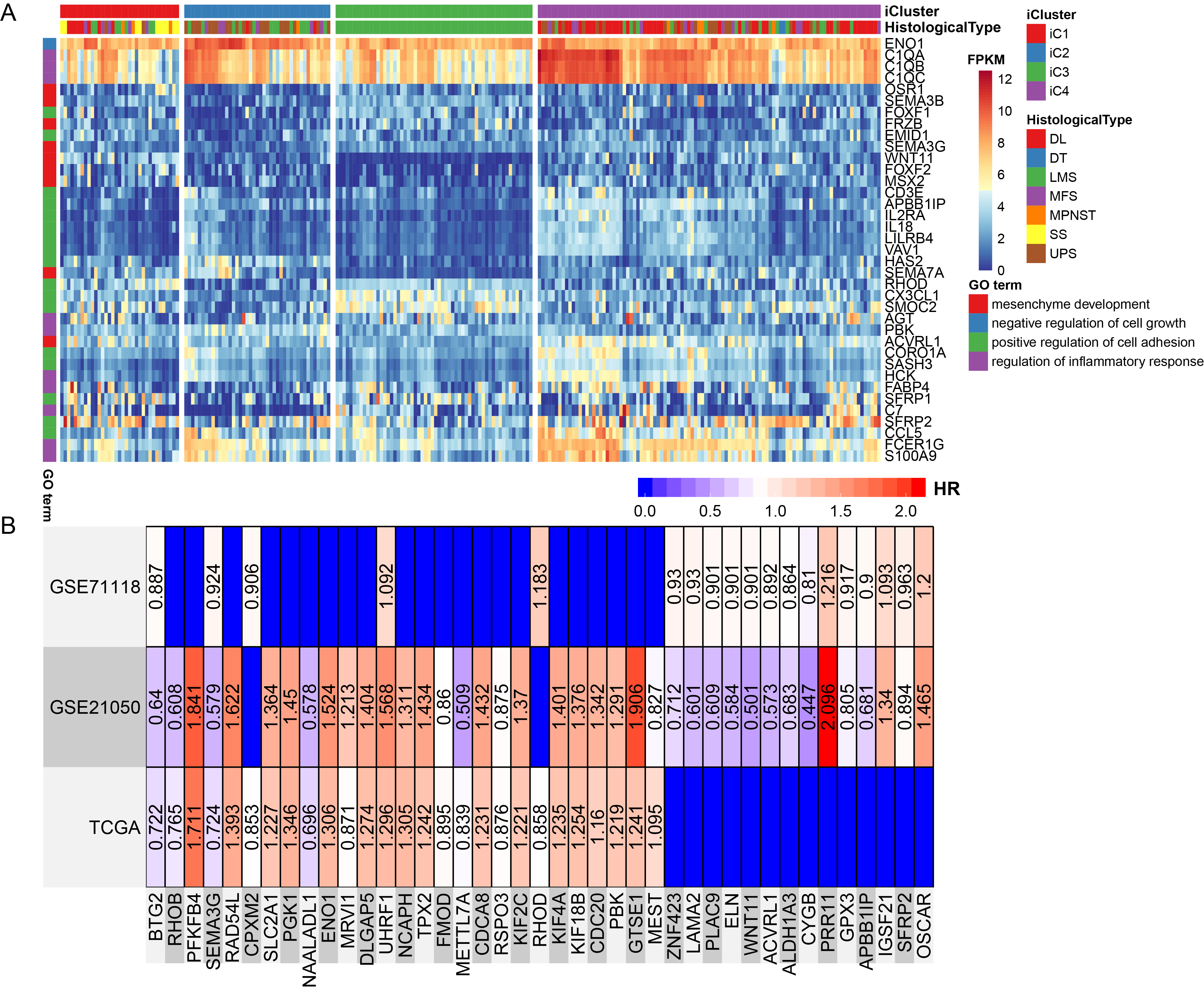

Supplement: Supplementary file 3 — Additional file 3: Supplementary Figure 3. Functional enrichment analysis and identification of differentially expressed genes. A: Heatmap of The GO enrichment analysis. B: Differentially expressed genes in three datasets. [file 12920_2021_876_MOESM3_ESM.tif]

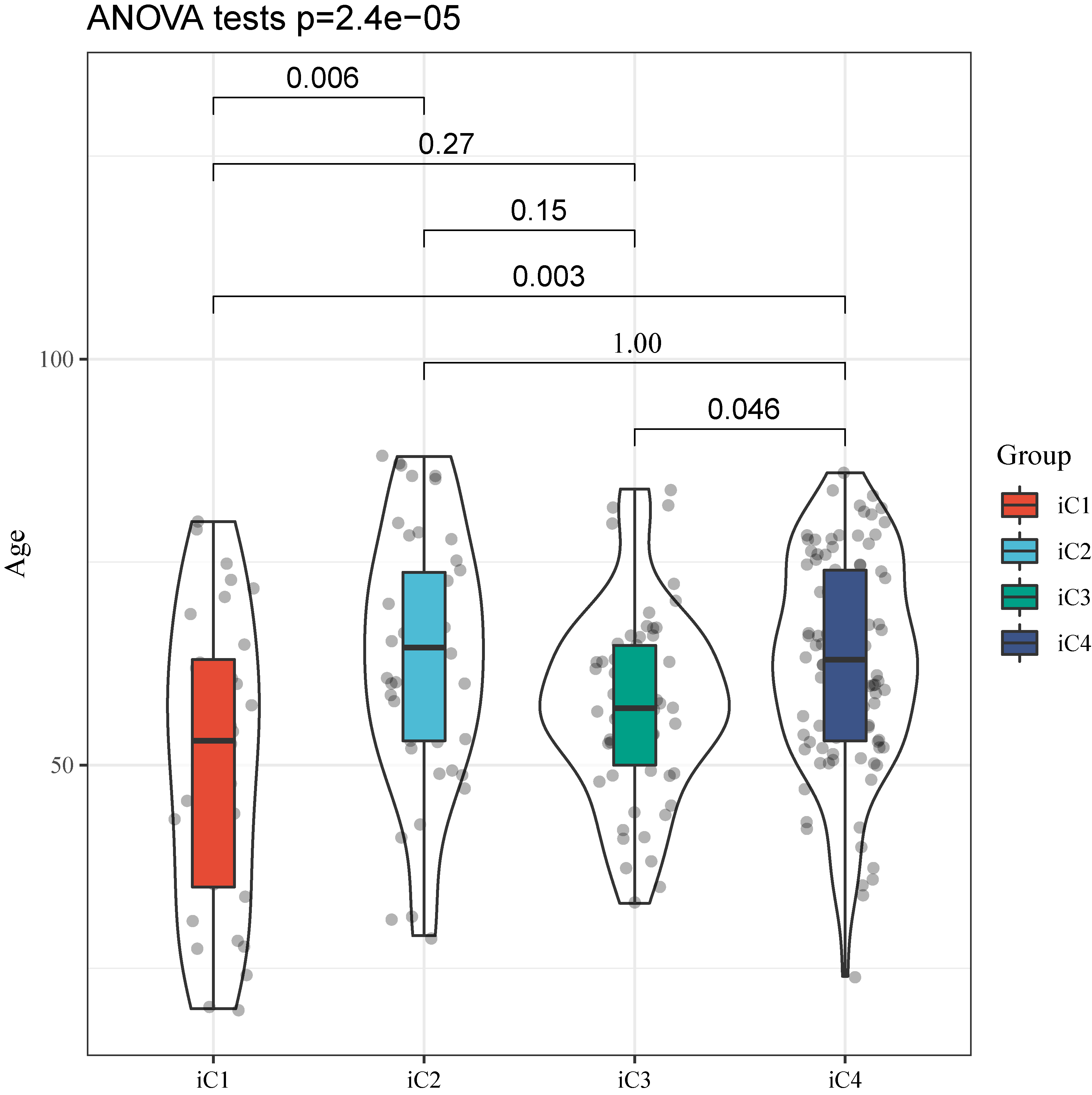

Supplement: Supplementary file 5 — Additional file 5: Supplementary Figure 5. Age in iC1 subgroup is significantly lower than iC2 and iC4, and age in iC3 is significantly lower than iC4. [file 12920_2021_876_MOESM5_ESM.tif]
